# Localized Quadratic RF Encoded Spin‐Echo With Spiral‐PRIME Reconstruction: A Practical Alternative to 3D FSE for High‐Resolution Volumetric Brain MRI

**DOI:** 10.1002/mrm.70476

**Published:** 2026-08-18

**Authors:** Guruprasad Krishnamoorthy, Julia V. Velikina, James G. Pipe

**Affiliations:** ^1^ Department of Radiology University of Wisconsin Madison Wisconsin USA

**Keywords:** denoising, spiral, sub‐mm isotropic t2w, whole brain volumetric imaging

## Abstract

**Purpose:**

To extend localized quadratic (LQ) RF encoded spin‐echo imaging with acquisition and reconstruction strategies that improve efficiency and artifact robustness, positioning it as a practical alternative to 3D FSE for high‐resolution volumetric brain MRI.

**Methods:**

The framework integrates (1) additional gradient‐echo readouts for simultaneous T2*w or PDw with T2w without prolonging scan time, (2) an in‐plane sampling scheme that distributes arms across shot/trajectory types to maximize k‐space coverage and render phase inconsistencies as incoherent residue, (3) sliding‐slice encoding to disperse through‐plane artifacts, (4) a novel loop‐ordering to avoid repeated startup cycles and improve motion robustness, and (5) a hybrid 2D/3D Physics‐based Reconstruction with Iterative Model‐based Enhancement (spiral‐PRIME) performing deblurring, fat–water separation, and 3D wavelet denoising. Healthy‐volunteer imaging (3 T) was compared with fully sampled LQ and conventional 3D FSE using peak signal‐to‐noise ratio, structural similarity index measure, pseudo‐replica SNR gain, and qualitative review.

**Results:**

Sliding‐slice with loop‐ordering reduced through‐plane artifacts and improved temporal efficiency, while in‐plane sampling dispersed trajectory inconsistencies as incoherent noise. Spiral‐PRIME suppressed undersampling artifacts and noise while preserving fine structure. Relative to spiral‐SENSE, spiral‐PRIME achieved consistent T2w SNR gains of ∼30%–40% in WM/GM and substantially higher gains for T2*w, with CNR improvements most pronounced for GM–WM. Despite *R* ≈ 2.33 undersampling, reconstructions closely matched fully sampled references and delivered quality comparable to or exceeding 3D FSE.

**Conclusion:**

LQ spin‐echo with spiral‐PRIME enables efficient, multi‐contrast volumetric brain imaging with robust artifact suppression and clinically meaningful SNR/CNR gains, supporting its potential as a practical alternative to 3D FSE.

## 1 Introduction

Obtaining T2‐weighted (T2w) images is an essential component of most brain MRI protocols. Two‐dimensional fast spin‐echo (2D FSE) remains the clinical workhorse for this purpose because of its speed and robustness. However, thin‐slice 2D FSE suffers from intrinsically low SNR efficiency owing to its slice‐by‐slice excitation scheme. To compensate, routine protocols typically employ relatively thick slices with high in‐plane resolution, acquired in multiple orientations. While this approach improves SNR, it prolongs overall scan time, reduces patient comfort, and introduces partial‐volume effects that can obscure subtle pathologies.

To overcome the inefficiency of 2D FSE, single‐slab 3D FSE [1, 2, 3] was introduced and is now implemented by all major vendors. These methods improve SNR efficiency, enable contiguous coverage and provide isotropic resolution with the flexibility of multiplanar reformats. Nevertheless, T2w 3D FSE relies on long echo trains to maximize temporal efficiency and minimize dead time within each TR. These long echo trains cause T2‐signal loss (which can also arise from T1 decay over echo trains on the order of T1) and associated blurring when this signal loss occurs primarily in the high spatial frequency part of k‐space. Also, due to the dependence of the T2 decay rate on flip angle in the small flip angle regime used by these methods [2], the spatial inhomogeneity of transmit RF power can result in spatially variable effective TE and associated T2 contrast. These effects may reduce to varying degrees the broader clinical utility of T2w 3DFSE.

Recently, localized quadratic (LQ) RF encoding, a hybrid 2D/3D encoding strategy with a spiral spin‐echo (SE) readout was proposed as an alternative approach for volumetric T2w imaging [4]. In LQ, a slab several times thicker than the prescribed slice thickness is excited using a RF pulse with a quadratic phase profile, and thin slices are reconstructed by removing the resulting quadratic phase from the k‐space data. This strategy combines the temporal efficiency of multi‐slice 2D acquisitions and SNR efficiency of 3D acquisitions. Furthermore, the SE design combined with long spiral readouts achieves temporal efficiency comparable to the long echo trains of 3D FSE, without the associated drawbacks of T2‐blurring and variable flip‐angle contrast. Nevertheless, long spiral readouts are sensitive to off‐resonance effects, necessitating advanced reconstruction strategies that continue to evolve and improve [5, 6, 7, 8, 9].

In this work, we extend the LQ SE technique through coordinated acquisition and reconstruction advances that enhance efficiency, robustness, and image quality. By integrating a gradient‐echo readout before the refocusing pulse, the framework recovers the “hallmark” feature of traditional 2D SE imaging, which is simultaneous proton‐density (PDw) and T2 weighting, and extends it into the volumetric domain with the addition of “free” PDw or T2*w contrasts. While clinical workflows often rely on separate gradient‐echo scans for iron and mineralization mapping, those acquisitions frequently suffer from T1 contamination due to the short repetition times (TR) required for throughput [10]. In contrast, our framework leverages the long TR of the LQ acquisition to provide inherently co‐registered, high‐purity contrasts in a single scan [4]. This facilitates the detection of paramagnetic iron complexes, such as ferritin and hemosiderin, that cause the rapid T2* dephasing necessary for diagnosing microhemorrhages without needing additional scans. To sample these contrasts efficiently, we implement an in‐plane strategy that distributes spiral arms across disparate echoes to eliminate redundancy, maximizing k‐space coverage while rendering trajectory inconsistencies into incoherent, high‐frequency residuals. These sampling benefits are augmented by a sliding‐slice encoding scheme that disperses through‐plane artifacts as noise‐like residues and a revised loop ordering that improves robustness to inter‐pass motion by placing the spiral arm in the outer loop. Finally, we introduce a hybrid 2D/3D spiral‐Physics‐based Reconstruction with Iterative Model‐based Enhancement (Spiral‐PRIME) reconstruction, which is a model‐based framework that performs joint spiral‐in/out deblurring and fat‐water separation while leveraging volumetric sparsity through 3D wavelet denoising to suppress undersampling artifacts and maximize SNR efficiency.

Some aspects of the pulse‐sequence innovations were previously presented in preliminary form at ISMRM [11, 12]. In this work, we introduce the novel reconstruction framework (spiral‐PRIME) and integrate it with the proposed acquisition strategies into a unified methodology. We provide a complete formulation and in vivo validation through whole‐brain imaging of healthy volunteers, with both qualitative and quantitative comparisons against fully sampled reconstructions and conventional 3D FSE.

## 2 Theory

This section outlines the theoretical foundation of the proposed framework. We first revisit LQ encoding and its SNR advantages, then describe pulse‐sequence extensions for efficient in‐plane and through‐plane sampling, and finally detail the spiral‐PRIME reconstruction model that integrates spiral deblurring, fat–water separation, and wavelet regularization. Together, these aspects provide high‐resolution volumetric brain MRI that is both efficient and practical.

### 2.1 LQ Encoding

A detailed analysis of LQ encoding has been presented in the foundational work by Pipe [13]. More recently, Kim et al. [4] incorporated LQ into T2w SE sequences, establishing a practical framework for volumetric SE imaging. Here, we provide only a brief introduction, emphasizing its sampling strategy and SNR benefits in the context of multi‐slice spin echo imaging with spiral readout.

LQ encoding modifies conventional slice excitation by using a rectangular‐magnitude RF pulse with a quadratic phase modulation. The LQ RF pulse can be written as

(1) $$b_{1}\left(t\right)=Ae^{-i\pi M{\left(\frac{t}{\tau }\right)}^{2}},-\tau <t<\tau$$

where *A* is the amplitude, *M* is the pulse time–bandwidth product (TBW), and τ is the pulse duration. For comparison, a conventional rectangular RF pulse of duration τ excites a slice of thickness (δ) which is defined by the full width at half maximum of the magnitude of the excitation profile

(2) $$\delta =\frac{1}{\gamma G\tau }$$

where γ is the gyromagnetic ratio and G is slice‐select gradient. In the LQ framework, successive slab excitations are shifted along the slice direction, and the spacing between these shifts must be equal to or smaller than the desired reconstructed slice resolution along z to ensure complete sampling of the thin‐slice information.

With quadratic phase modulation, the RF bandwidth increases to *M/* τ, increasing the slab thickness to *M* δ. Formally, when an RF pulse $b_{1}\left(t\right)$ excites magnetization f(z) along the slice direction z, the measured signal is the convolution of f(z) with the slice profile h(z):

(3) s(z)=f(z)⨂h(z)

For a quadratic‐phase RF pulse, the slice profile h(z) corresponds to a slab of width *M* δ. A stack of slices can be Fourier transformed into the slice‐frequency domain, giving

(4) S(k)=F(k)⨂H(k).

The shape of H(k) is comparable to that of the RF pulse (constant amplitude with a quadratic phase). This quadratic phase can be removed from S(k), yielding the desired thin‐slice profile comparable to *1/*, but with an SNR increase of $\sqrt{M}$.

The observed SNR increase is fundamentally tied to the global excitation profile of the LQ framework. Because all spins within the imaging volume are excited in each TR and contribute to the acquired signal, LQ encoding provides SNR advantages analogous to 3D imaging, yet it retains the temporal efficiency of multi‐slice 2D acquisitions. Crucially, unlike conventional 3D strategies, the LQ framework resolves thinner slices from the excited slabs through the known phase profile of the LQ RF pulse rather than discrete phase‐encoding loops. Furthermore, the spiral readout offers sampling efficiency comparable to the long echo trains of 3D‐FSE but avoids the associated T2 blurring, (potentially) high SAR, and stimulated echo contamination.

### 2.2 Pulse Sequence Framework

The proposed sequence builds on the fundamental LQ SE framework described previously [4]. Excitation was performed using a rectangular‐magnitude RF pulse with a 90° flip angle, modulated with a quadratic phase profile to achieve LQ encoding. For refocusing, a conventional sinc (TBW 6.4) 180° RF pulse was used. A spiral in–out trajectory measured T2W data after refocusing, and an optional spiral readout was inserted before the refocusing pulse to simultaneously provide gradient‐echo contrast, as shown in Figure 1a.

**FIGURE 1:**
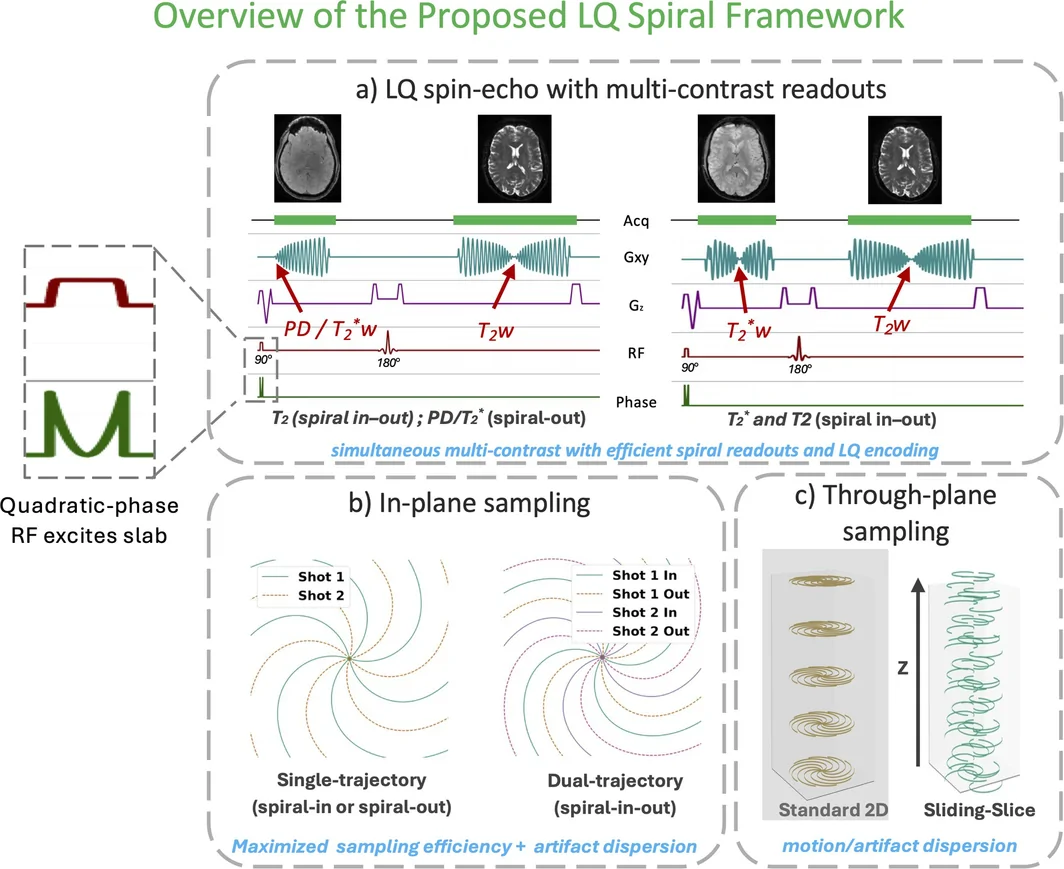
Overview of the proposed localized quadratic (LQ) spin‐echo framework with simultaneous multi‐contrast readouts and efficient sampling strategies. A quadratic‐phase RF pulse excites a thick slab, enabling (a) LQ spin‐echo acquisition with spiral readouts that provide flexible contrast options (PD/T2*/T2) without prolonging scan time. The slice‐select gradient architecture utilizes a five‐lobe design to achieve zeroth and first gradient moment compensated selection while providing selective refocusing and robust FID crushing of the 180° RF pulse. (b) In‐plane sampling maximizes k‐space coverage efficiency and disperses trajectory phase inconsistencies as incoherent high‐frequency artifacts. (c) Through‐plane sampling with sliding‐slice renders motion‐ and slice‐boundary artifacts incoherent compared to conventional 2D multi‐slice imaging. Together, these design elements provide robust, efficient volumetric imaging with simultaneous multi‐contrast capability.

#### 2.2.1 In‐Plane Sampling and Arm‐Order

The acquisition uses two interleaved shots, each containing a set of uniformly spaced spiral arms. The shots are acquired with a small echo‐time shift to enable spectral fat–water separation during reconstruction. Within each shot, the angular position of the $i^{\mathrm{th}}$ spiral arm is uniformly spaced as

(5) $$∆\theta =\frac{2\pi }{N_{\mathrm{arm}}},\theta_{i}=i\cdot ∆\theta ,i=0,1,\ldots ,N_{\mathrm{arm}}-1$$

where $N_{\mathrm{arm}}$ is total arms per slice per shot. The order in which these uniformly spaced arms are traversed is determined by a pseudo‐golden‐angle scheme [14], ensuring uniform k‐space coverage across TRs and robustness to motion and temporal system instabilities. To prevent redundant k‐space sampling of spiral in‐out readouts, the spiral‐out ($\theta_{\text{out}}$) arm is rotated relative to its paired spiral‐in ($\theta_{\text{in}}$) arms as

(6) $$\theta_{\text{out}}=\theta_{\text{in}}+\pi \left(1+\frac{1}{N_{\mathrm{arm}}.N_{\text{shot}}}\right)$$

where $N_{\text{shot}}$ is the number of shots. These multiple shots are echo‐shifted to facilitate robust spectral fat‐water separation in the reconstruction. To prevent redundancy across shots, additional angular offsets [11] are applied:

(7) $$\theta_{T,\text{shot}}=\theta_{i}+m\left(\frac{∆\theta }{N_{\text{shot}}}\right),m=0,1,\ldots ,N_{\text{shot}}-1,T\in \left\{\text{in},\text{out}\right\}$$

This combined sampling strategy is designed to provide two primary advantages: (1) it maximizes k‐space sampling efficiency by ensuring that complementary trajectories do not redundantly sample the same regions, and (2) by placing these trajectories' data in close angular proximity in k‐space, any phase inconsistencies in the acquired data between shots or trajectory types such as those arising from eddy currents are shifted into high‐frequency modulations. Consequently, these inconsistencies manifest in the image domain as incoherent, noise‐like artifacts rather than structured ghosting, which is expected to improve the perceived image quality and diagnostic reliability.

#### 2.2.2 Through‐Plane Sampling and Slice‐Order

In conventional 2D multi‐slice imaging, multiple spiral arms (or Cartesian phase‐encoding lines) are acquired at a fixed slice location (*z*‐axis frequency offset). In contrast, the sliding‐slice approach acquires each spiral arm at a unique *z*‐axis position (Figure 1c), originally developed for time‐of‐flight imaging [15]. Here, we adapt sliding‐slice for SE acquisitions to disperse through‐plane artifacts incoherently along the *z*‐axis, reducing their visual prominence.

In conventional 2D acquisitions, organizing slices into passes requires repeated startup cycles to reach steady state, which reduces efficiency and introduces inter‐pass inconsistencies. The proposed LQ framework instead excites contiguous slabs across the entire volume every TR, enforcing a single global steady state. Since magnetization remains stable throughout the volume, slice grouping is unnecessary, allowing the spiral arm index to serve as the outermost loop, shown in Figure 2. This ordering improves efficiency by requiring startup cycles only once at the scan's start. Furthermore, distributing slice positions across spiral arms rather than passes enhances motion robustness by rendering through‐plane artifacts incoherent along the slice direction. We therefore expect higher sampling efficiency, improved multiplanar reformat reliability, and reduced slice‐direction discontinuities compared to standard ordering.

**FIGURE 2:**
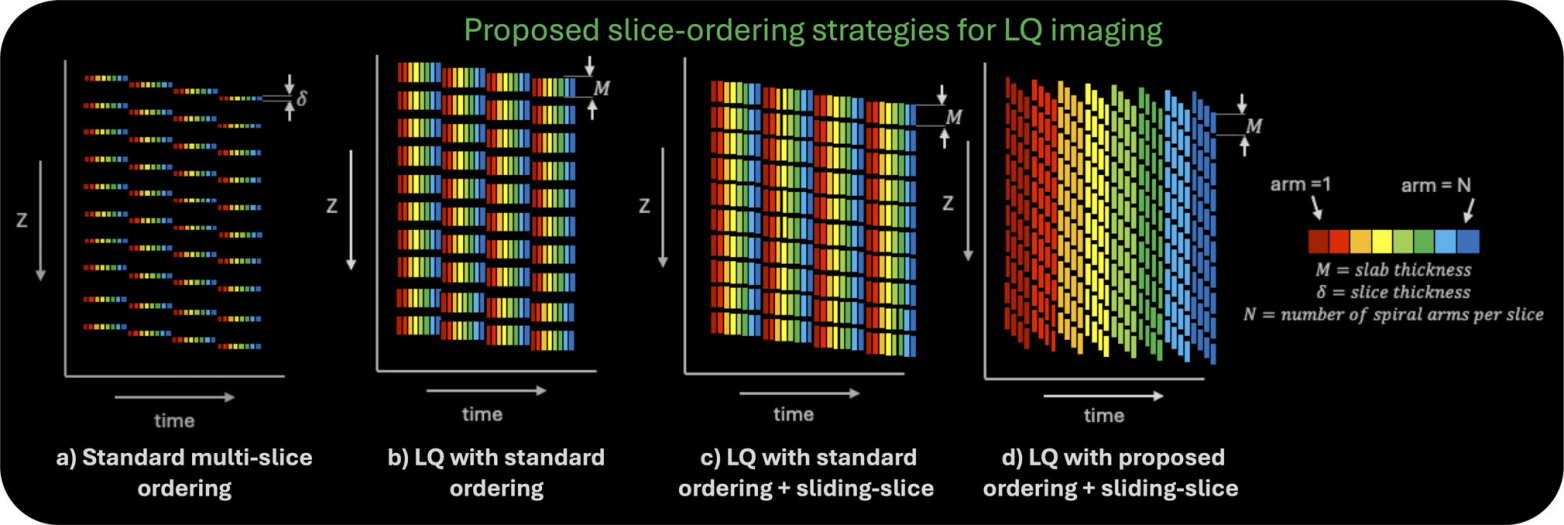
Illustration of acquisition loop ordering for standard multi‐slice 2D (a) and localized quadratic encoding (LQE, b–d). (a) Standard multi‐slice 2D ordering, where each pass collects multiple slices and requires startup cycles. (b) LQE with standard slice ordering. (c) LQE with sliding‐slice ordering, where each spiral arm samples a slightly shifted slice location along the *z*‐axis. (d) Proposed LQE ordering with sliding‐slice, in which spiral arms form the outermost loop, dispersing slice/arm acquisition across TRs and eliminating repeated startup cycles. In this example, 40 slices are acquired in 4 passes, with 10 slices per pass and 8 spiral arms (Kxy) per slice.

### 2.3 Reconstruction Framework

The aim of the proposed reconstruction framework is to jointly address the key challenges of spiral acquisitions, namely off‐resonance, chemical‐shift–related blurring and undersampling artifacts, while further improving the SNR efficiency. To this end, we developed a unified model‐based approach that performs spiral deblurring, fat–water separation, parallel imaging, and 3D wavelet‐based noise suppression within an Alternating Direction Method of Multipliers (ADMM) optimization [16]. We refer to this hybrid 2D/3D integrated framework as spiral‐PRIME reconstruction (Physics‐based Reconstruction with Iterative Model‐based Enhancement).

The forward model was adapted from Chao et al. [9], (Model 3). For coil c, shot index $m=0,1..,N_{\text{shot}}-1$ with $N_{\text{shot}}$ represent total shots acquired the acquired data are modeled as:

(8) $$d_{c,m}=F_{m}\left\{\sum_{i=w,f}C_{c}\left(\vec{r}\right)\left[x_{i}\left(\vec{r}\right)⊛\Phi_{\vec{r}}\left(\vec{r},m\right)\right]⊛\Omega_{i}\left(m\right)\right\}$$

where $F_{m}$ is the gridding operator mapping images to spiral samples, $C_{c}$ is the coil sensitivity map, $x_{i}$ denotes water (w) and fat (f) images, $\Phi_{\vec{r}}$ represent the spatially varying point spread function (PSF) capturing off‐resonance, $\Omega_{i}$ represent the PSF of the average off‐resonant frequency including chemical shift of the entire image, $\vec{r}$ represent 2D spatial coordinates and ⊛ is convolution operator.

The image reconstruction was then formulated as an inverse problem with $\ell_{1}$ regularization [17]:

(9) $$\underset{x}{\min\frac{1}{2}}\left\|\tilde{E}x-d\right\|\begin{array}{c} 2\\ 2 \end{array}+\sum_{c\in \left\{w,f\right\}}\lambda_{c}{\left\|\Psi x_{c}\right\|}_{1}$$

where $x=\left[x_{w},x_{f}\right]\in {\mathbb{C}}^{N}$ contains unknown water and fat images, Ψ is a 3D wavelet transform, $\lambda_{c}$ are regularization weights learned for each species. The forward operator $\tilde{E}$ was defined as

(10) $$\tilde{E}=\left[\begin{array}{l} E_{\text{out}}\\ E_{\text{in}} \end{array}\right],d=\left[\begin{array}{l} d_{\text{out}}\\ d_{\text{in}} \end{array}\right]$$

When both spiral‐in and spiral‐out data are acquired, $\tilde{E}$ contained both forward models and data were concatenated accordingly. For single‐trajectory acquisitions, only the corresponding operator ($E_{\text{out}}$ or $E_{\text{in}}$) and data were retained. Regularization was applied independently to water and fat images using wavelet $\ell_{1}$ norms. Soft‐thresholds were estimated using SureShrink [18, 19], but adapted to complex MRI under a Rayleigh‐based noise estimate (median/1.177) instead of the standard Gaussian assumption. To mitigate the shift‐variance of discrete wavelet transforms and reduce pseudo‐Gibbs artifacts, cycle spinning [19, 20] was applied.

Spiral‐PRIME employs a hybrid 2D/3D reconstruction, solving the inverse problem (Equation 6) via the ADMM, detailed in the Appendix A. Each iteration includes: (1) a slice‐wise conjugate gradient (CG) projection to enforce data consistency, enabled by the separability of the LQ and sliding‐slice forward model; (2) volumetric 3D wavelet denoising to exploit sparsity and suppress noise; and (3) volumetric dual updates to balance fidelity and regularization. The CG step effectively handles the ill‐conditioned forward operator resulting from coil sensitivities, gridding, B0 inhomogeneity, and fat–water separation. The overall reconstruction framework is illustrated in Figure 3.

**FIGURE 3:**
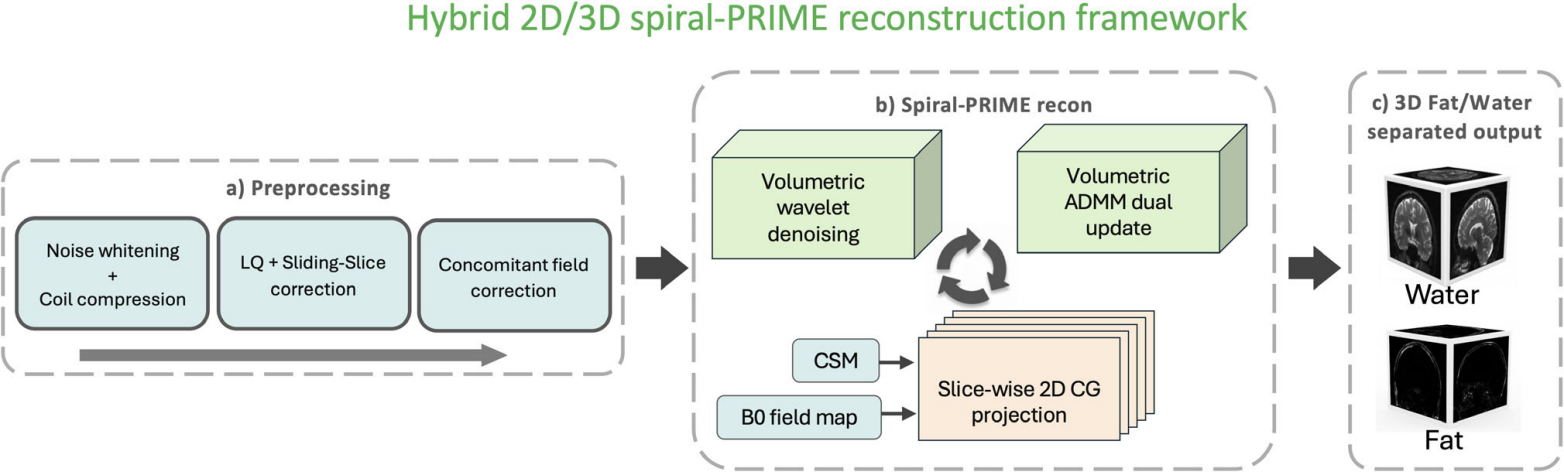
Schematic of the proposed spiral‐PRIME reconstruction framework. (a) Raw k‐space data are preprocessed with noise whitening, coil compression, localized quadratic (LQ) encoding, sliding‐slice acquisition, and concomitant field correction. (b) Image reconstruction is performed using an ADMM‐based iterative loop. The projection step enforces slice‐wise data consistency via conjugate gradient (CG) using coil sensitivity maps (CSM) and B_0_ field maps, while the 3D denoising and dual update steps act on the full volume to suppress noise, mitigate slice‐wise inconsistencies, and exploit inter‐slice sparsity. (c) The final outputs are separate 3D water and fat images. Spiral‐PRIME, spiral‐Physics‐based Reconstruction with Iterative Model‐based Enhancement.

## 3 Methods

In vivo experiments were conducted on five healthy volunteers under an institutionally approved and consented protocol using a 3 T GE Signa Premier scanner (GE Healthcare, USA) with a 16‐channel head and neck coil. Field maps were obtained using a spiral‐based sequence [21]. Coil sensitivity maps and noise covariance were estimated from a short prescan. Spiral gradient waveforms, k‐space trajectories, and density‐compensation weights were generated using analytical formulations as described previously [22], with vendor‐provided gradient pre‐emphasis used to minimize eddy‐current distortions. All image reconstructions for LQ were carried out offline using GPI platform [23] on an Apple MacBook Pro equipped with an M2 Pro processor (12‐core CPU, 64 GB RAM). The slice‐wise conjugate‐gradient step was implemented in C++ with multi‐threading across slices, while volumetric denoising and ADMM updates were implemented in Python. The complete reconstruction code and representative sample data are openly available at https://github.com/gpilab/spiral‐prime.

### 3.1 Simultaneous T2*w and T2w Imaging Under Different Slice‐Ordering Strategies and In‐Plane Sampling Pattern

To evaluate the effect of slice‐ordering, whole‐brain simultaneous T2w and T2*w imaging was performed using the proposed sequence under both conventional and sliding‐slice ordering (Figure 2b,c). Imaging parameters for this experiment correspond to the “1 mm‐T2w/T2*w” protocol in Table 1. To ensure that observed differences arose solely from the acquisition strategy, all datasets were reconstructed using the joint spiral deblurring and fat‐water separation forward model described in Equation (8). This system was solved via the CG method without any regularization. Depending on the acquisition, both single‐trajectory and dual‐trajectory reconstructions were implemented. This linear reconstruction framework is referred to as spiral‐SENSE throughout the article.

**TABLE 1:** Imaging parameters for localized quadratic (LQ) spiral spin‐echo and standard 3D fast spin‐echo (3D FSE) acquisitions.

Sequence	LQ spin‐echo				3D fast spin‐echo	
Protocol	1 mm‐T2w/T2*w	FS‐T2w/T2*w	US‐T2w/T2*w	US‐T2w/PDw	Longer‐3DFSE	Shorter‐3DFSE
Scan orientation	Axial					
FOV	24 × 24 × 16 cm^3^	24 × 24 × 18 cm^3^				
Resolution	1.0 × 1.0 × 1.0 mm^3^	0.8 × 0.8 × 0.8 mm^3^				
Spin‐echo k‐space trajectory/readout duration	Spiral in‐out/15–15 ms	Spiral in‐out/20–20 ms			Cartesian	
Gradient‐echo k‐space trajectory/readout duration	Spiral in‐out/15–15 ms	Spiral out/20 ms			NA	
TE/TR Spin‐echo	90/3000 ms	80/3000 ms				
TE/TR Gradient‐echo (read before refocusing)	22/3000 ms	11/3000 ms		6/3000 ms	NA	
Bandwidth (kHz)	±250 kHz				±62.5 kHz	
Number of arms	14		6		NA	
LQ time‐bandwidth‐product (M)	7	9			NA	
No. of shots/echo‐shift	2/1.19 ms				1	
Echo train length/Echo spacing	NA				190/4.53 ms	190/4.53 ms
Acceleration factor	In‐plane: 1.0 Slice: 1.0	In‐plane: 1.0 Slice: 1.0	In‐plane: 2.33 Slice: 1.0		ASSET: Phase = 2.0 ASSET: Slice = 1.32	ASSET: Phase = 2.0 ASSET: Slice = 2.0 HyperSENSE: 1.4
NEX	1				2	
Scan time	10′00″ to 11′24″	12′48″	5′36″		12′52″	5′34″
Notes	a. ASSET: Vendor implementation of SENSE‐based parallel imaging. b. HyperSENSE: Vendor implementation of compressed sensing. c. 3D FSE TE: The 80‐ms TE represents the contrast‐equivalent value, not the center of k‐space acquisition time. d. Fat‐Water Separation: LQ spin‐echo utilizes a dual‐shot interleaved strategy (1.19‐ms echo‐time shift across separate TRs). This interleaving provides the necessary phase evolution for robust separation at 3 T. e. NEX Selection: On the 3 T GE Signa Premier, axial 3D FSE is vendor‐restricted to even NEX values (minimum NEX = 2) to ensure echo‐train stability.					

In addition, the in‐plane sampling strategy was tested by retrospectively undersampling fully sampled datasets. Reconstructions were compared between (i) uniform undersampling, where the same spiral arms were selected across all shots and trajectories, and (ii) the proposed in‐plane scheme, where different arms were distributed across shots and trajectories to maximize k‐space coverage and disperse inconsistencies as incoherent high‐frequency noise. Both strategies were implemented at an acceleration factor of *R* = 3 and reconstructed using spiral‐SENSE for direct comparison with the fully sampled reference.

### 3.2 Spiral‐PRIME Reconstruction Parameter Tuning

Two fully sampled whole‐brain datasets from different subjects were acquired using the “FS‐T2w/T2*w” protocol (18 spiral arms per shot instead of 14 arms) and reconstructed with spiral‐SENSE as ground‐truth references. Data were retrospectively undersampled (9, 6, 4 arms) across spiral‐in/out and shot dimensions to mimic the sampling scheme in Figure 1b. ADMM reconstructions tested penalty initializations (*ρ*_0_ = 0.5–4.0, *γ* = 1.1) and growth factors (γ = 1.0–1.5, *ρ*_0_ = 1.0). Convergence was monitored by the residual, defined as the $\ell_{2}$‐norm difference between successive image updates (stop if ‖*x* ^*k*^ − *x* ^*k*−1^‖_2_/‖*x* ^*k*−1^‖_2_ < 10^−6^), and reconstruction quality was assessed using peak signal‐to‐noise ratio (PSNR) and structural similarity index (SSIM) relative to the spiral‐SENSE reference. Wavelet denoising was evaluated using 2D (slice‐wise SureShrink) or 3D (volumetric SureShrink) wavelet transforms using Daubechies 5 wavelet system. Reconstructions ran for up to 20 iterations or until convergence. Optimal ADMM parameters were determined based on PSNR and SSIM across undersampling levels.

### 3.3 SNR Gain Estimation

SNR gain achieved by spiral‐PRIME was evaluated using a pseudo‐replica framework [24] originally developed for linear reconstructions. Because spiral‐PRIME incorporates nonlinear regularization (wavelet denoising within ADMM), absolute SNR cannot be defined under the same assumptions; therefore, our analysis focused on estimating relative SNR gain compared to the (linear) spiral‐SENSE reconstruction. Multiple instances of complex, correlated noise, consistent with measured coil noise variance, were added to multi‐coil k‐space data. To ensure consistent nonlinearity across replicas, soft thresholds for wavelet denoising were precomputed from the adjoint reconstruction without noise addition and then fixed for all subsequent replicas. Multiple replicas (*N* = 16) were reconstructed with both pipelines using identical parameters. Noise was added at amplitudes orders of magnitude (×0.1) smaller than the measured noise so that ensemble variability reflected noise sensitivity without altering the underlying reconstruction solution. We verified that decreasing/increasing the added‐noise scale (×0.05–×0.2) did not materially change the gain estimates, indicating the reconstruction operated in a locally linear regime around the solution despite the global nonlinearity. Voxel‐wise SNR was computed as the ratio of the mean to the standard deviation across replicas, and SNR gain maps as follows:

(11) $$\mathrm{SNR}\;\text{gain}\left(\%\right)=100*\left(\frac{{\mathrm{SNR}}_{\text{spiral}-\text{DEFT}}}{{\mathrm{SNR}}_{\text{spiral}-\text{SENSE}}}-1\right)$$

where ${\mathrm{SNR}}_{\text{spiral}-\text{DEFT}}$ and ${\mathrm{SNR}}_{\text{spiral}-\text{SENSE}}$ are SNR obtained with spiral‐PRIME and spiral‐SENSE reconstruction respectively. To further support the estimates, mean and variance maps from the replica ensembles were visualized. In addition, ROI‐based SNR gains were computed in white matter (WM), gray matter (GM), Cerebrospinal fluid (CSF), and fat to provide quantitative regional measures of improvement. Complementary to SNR, contrast‐to‐noise ratio (CNR) was also evaluated for diagnostically relevant tissue pairs (WM–GM, WM–CSF, and GM–CSF). CNR was defined as the difference in mean signal between two ROIs divided by the average noise standard deviation within those ROIs, and CNR gain was reported as the percentage improvement of spiral‐PRIME over spiral‐SENSE.

Finally, fully sampled datasets corresponding to “FS‐T2w/T2*w” in Table 1 were reconstructed using spiral‐SENSE and visually compared against undersampled dataset corresponding to “US‐T2w/T2*w” protocol reconstructed using spiral‐PRIME to confirm that the estimated SNR gains were consistent with perceptible improvements in image quality.

### 3.4 Visualizing Inherent Co‐Registration of Multi‐Contrast Acquisitions

For simultaneous acquisitions, the protocols listed as “US‐T2w/T2*w” and “US‐T2w/PDw” in Table 1 were used. Each contrast was reconstructed independently using the same reconstruction pipeline. For visualization, the resulting volumes were overlaid as color composites (red = T2w, green = PDw; red = T2w, blue = T2*w) to illustrate the inherent spatial co‐registration of the contrasts, without the need for retrospective alignment.

### 3.5 Comparison of the Proposed LQ Framework With Standard 3D FSE for High‐Resolution Volumetric T2w Imaging

To assess the capability of the proposed framework for high‐resolution volumetric imaging, 800 μm isotropic T2w datasets were acquired using both 3D FSE and LQ SE protocols, with acquisition parameters matched where possible (Table 1). Two reference 3D FSE datasets were obtained using the vendor's reconstruction; one accelerated with ASSET (linear Cartesian Sense) alone, and a second shorter scan accelerated with combined ASSET and HyperSense (nonlinear Cartesian Compressed‐Sensing). Image reconstruction for 3D FSE was performed directly on the scanner using vendor reconstruction. LQ datasets were acquired with spiral‐SENSE and spiral‐PRIME reconstructions at comparable scan times. Axial, coronal, and sagittal reformats were generated for each protocol. Representative regions were selected for inset comparison to illustrate lesion conspicuity, cortical delineation, cerebellar detail, and areas of residual B0 sensitivity, enabling direct evaluation of structural fidelity between the proposed LQ framework and standard 3D FSE.

## 4 Results

Figure 4 shows brain images acquired with the proposed LQ sequence, demonstrating simultaneous T2w and T2*w imaging under different slice‐ordering strategies (Figure 2). In conventional multi‐slice 2D acquisitions, interleaved slice ordering produced discontinuities in the slice direction when motion occurred between passes (Figure 4a). The sliding‐slice technique reduced the conspicuity of these artifacts by dispersing them incoherently, though some residual structure remained (Figure 4b). When sliding‐slice was combined with the proposed acquisition loop ordering, through‐plane artifacts and motion sensitivity were further suppressed, yielding images with continuity and uniformity that closely resembled 3D acquisitions while also reducing total scan time (Figure 4c). Figure S1 further demonstrates the effect of the proposed in‐plane sampling strategy: distributing spiral arms across shots and trajectory directions disperses undersampling artifacts as incoherent noise, most prominently visible in the fat channel.

**FIGURE 4:**
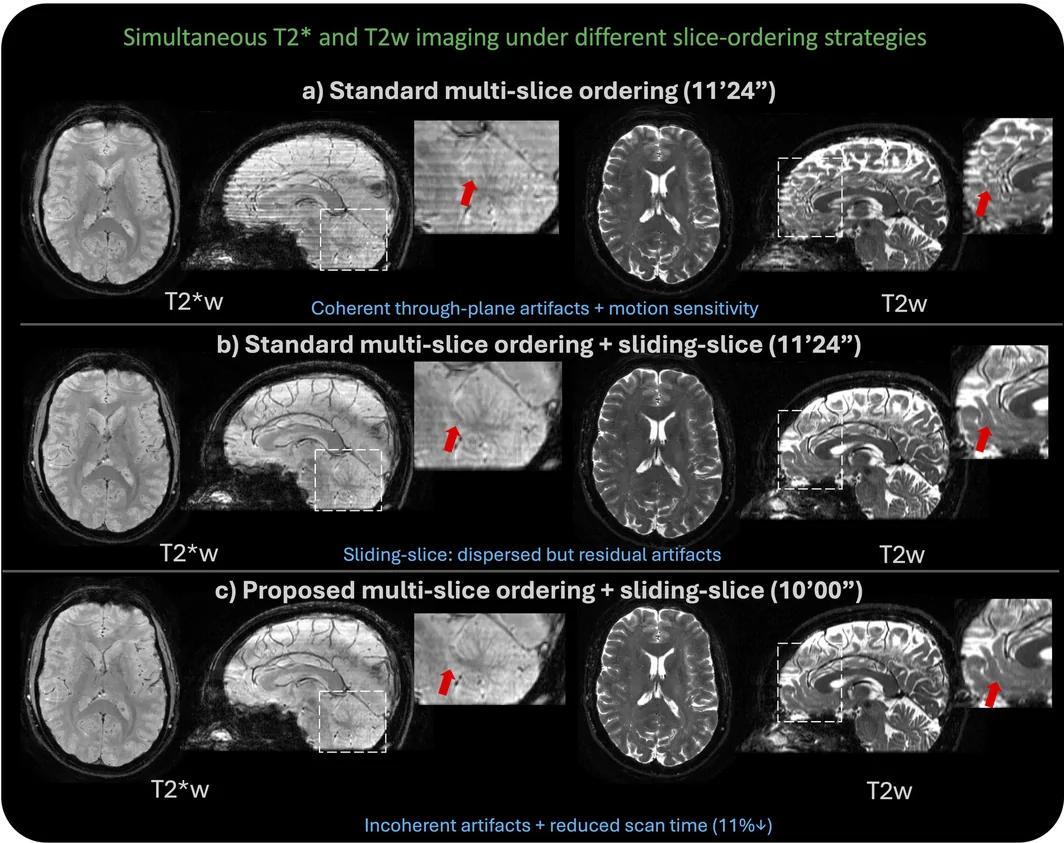
Simultaneous T2*‐ and T2w imaging with the proposed LQ sequence. (a) Standard multi‐slice ordering produces coherent through‐plane artifacts (red arrows) and strong sensitivity to inter‐pass motion. (b) Incorporating sliding‐slice disperses these artifacts across slices, reducing their conspicuity but leaving residual structure. (c) The proposed strategy combines sliding‐slice with optimized loop ordering, which both renders through‐plane artifacts incoherent and mitigates the appearance of motion artifacts. In addition, the proposed ordering eliminates repeated startup cycles before each pass, yielding shorter overall scan times while maintaining the temporal efficiency of multi‐slice 2D. Insets highlight regions where artifact mitigation is most evident.

The influence of ADMM parameters on reconstruction is summarized in Figure S2. Reconstructions with small penalty initialization (*ρ*_0_ < 1.0) over‐enforced data consistency and degraded image quality, while higher values (*ρ*_0_ > 2.0) slowed convergence. Moderate initialization (*ρ*_0_ ≈ 1.0) with mild growth (*γ* ≈ 1.1) provided the best trade‐off, stabilizing within 6 iterations, after which reconstructions were terminated when the residual fell below 1e^−4^. Quantitative PSNR and SSIM values across different acceleration factors and trajectory types are reported in Table S1, confirming the robustness of this parameter choice.

Figure S3 evaluates the effect of wavelet dimensionality in the denoising step. Compared with spiral‐SENSE, slice‐wise (2D) wavelet denoising reduced noise but introduced slice‐dependent artifacts and irregularities. In contrast, 3D wavelet denoising achieved consistent noise suppression across slices, preserved structural fidelity, and avoided spurious slice‐level artifacts, confirming the advantage of volumetric denoising within the spiral‐PRIME framework.

Figure 5 compares spiral‐SENSE and spiral‐PRIME reconstructions on a 2.3× undersampled T2w dataset against a fully sampled spiral‐SENSE reference. Spiral‐PRIME suppresses noise and undersampling artifacts while preserving fine structural detail, yielding image quality more consistent with the fully sampled reference. Figure 6 extends this comparison to the simultaneously acquired T2*w dataset, demonstrating similar improvements.

**FIGURE 5:**
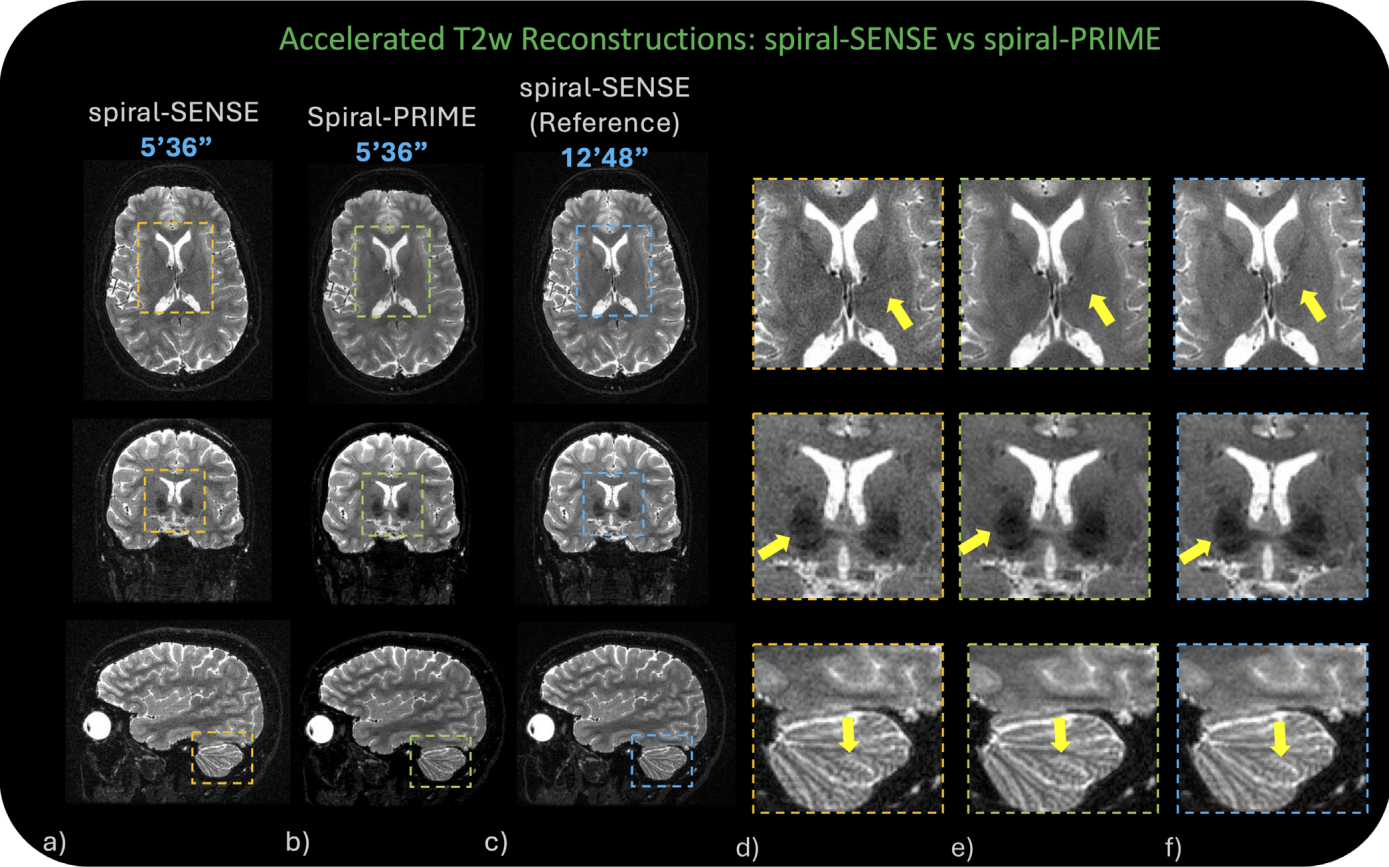
Comparison of accelerated reconstructions on a T2w dataset with spiral‐SENSE and spiral‐PRIME against a fully sampled reference. Axial, coronal, and sagittal views reconstructed with (a) spiral‐SENSE reconstruction (5′36″), (b) the proposed spiral‐PRIME method on the same dataset as (a), and (c) fully sampled spiral‐SENSE (12′48″) which is a different dataset. Corresponding zoomed insets are shown in (d–f) for regions marked by dashed boxes. Yellow arrows indicate areas where spiral‐PRIME suppresses noise and undersampling artifacts while maintaining structural resolution, yielding image quality more consistent with the fully sampled reference.

**FIGURE 6:**
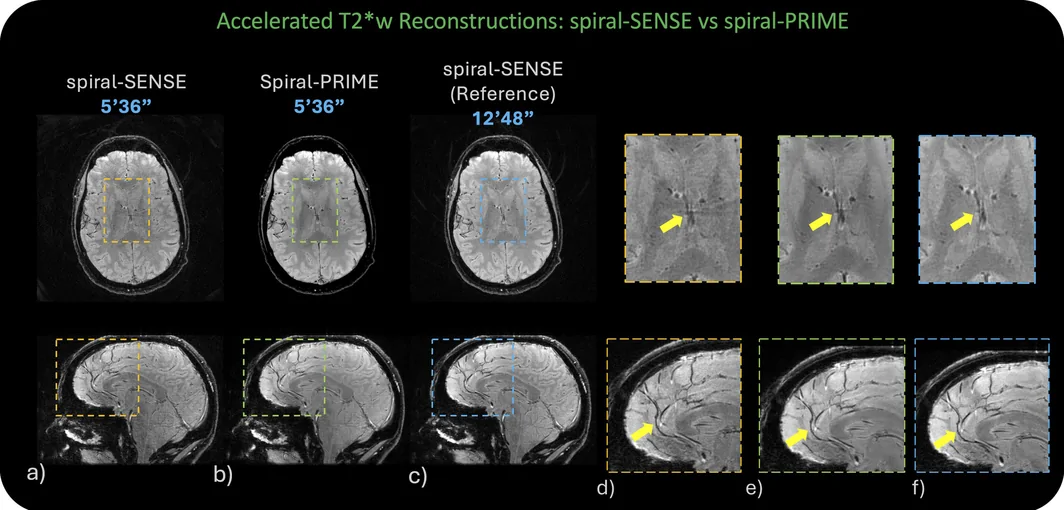
T2*w images acquired from the same scan shown in Figure 5, obtained by inserting a spiral‐out readout before the refocusing pulse (TE = 11 ms) to provide additional T2*w contrast without increasing scan time. Axial (top row) and sagittal (bottom row) views reconstructed with (a) spiral‐SENSE from a 5′36″ acquisition, (b) spiral‐PRIME from the same 5′36″ data, and (c) fully sampled spiral‐SENSE from a 12′48″ acquisition. Corresponding zoomed insets (right) highlight venous conspicuity and cortical detail (d–f). Yellow arrow indicates Spiral‐PRIME suppresses undersampling artifacts and noise relative to spiral‐SENSE while preserving venous darkening and fine structure, yielding image quality closely matching the fully sampled reference.

Figure 7 summarizes SNR and CNR gains with spiral‐PRIME versus spiral‐SENSE. Voxel‐wise SNR gain maps show widespread improvements, especially in WM and GM; across subjects, WM/GM SNR increases are up to 40% for T2w and up to 100% T2*w, with smaller gains in CSF and fat. CNR also improves across GM–WM, CSF–WM, and CSF–GM. Figure S4 provides pseudo‐replica mean/variance maps, confirming unchanged mean anatomy but reduced variance with spiral‐PRIME.

**FIGURE 7:**
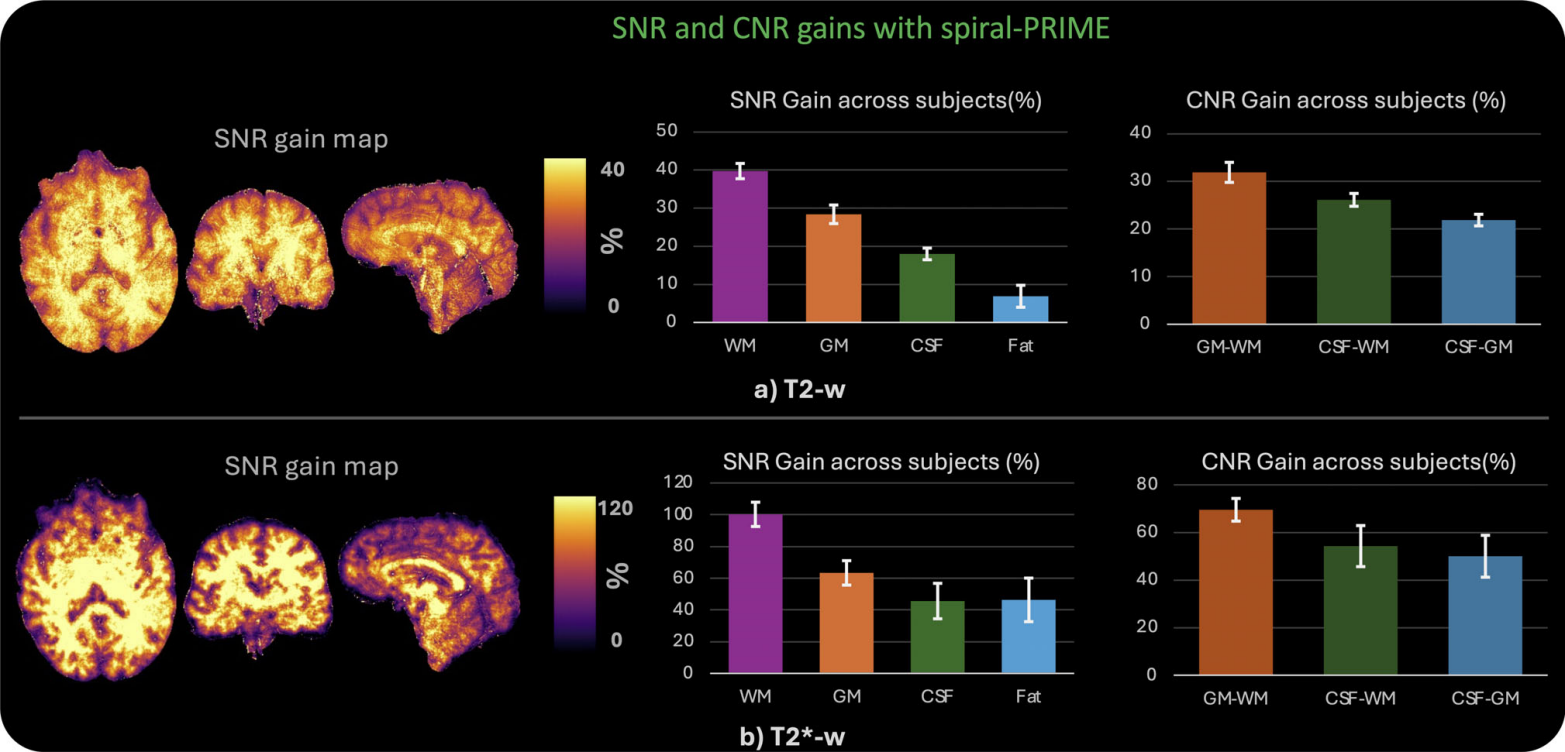
SNR and CNR gains with spiral‐PRIME. Voxel‐wise maps (left) and across‐subject analyses (middle, right) demonstrate improved SNR efficiency and tissue contrast for both (a) T2w and (b) T2*w imaging compared to spiral‐SENSE.

Figure 8 highlights the inherent co‐registration of contrasts achieved with the proposed sequence. The overlays demonstrate perfect alignment of tissue boundaries without the need for retrospective registration.

**FIGURE 8:**
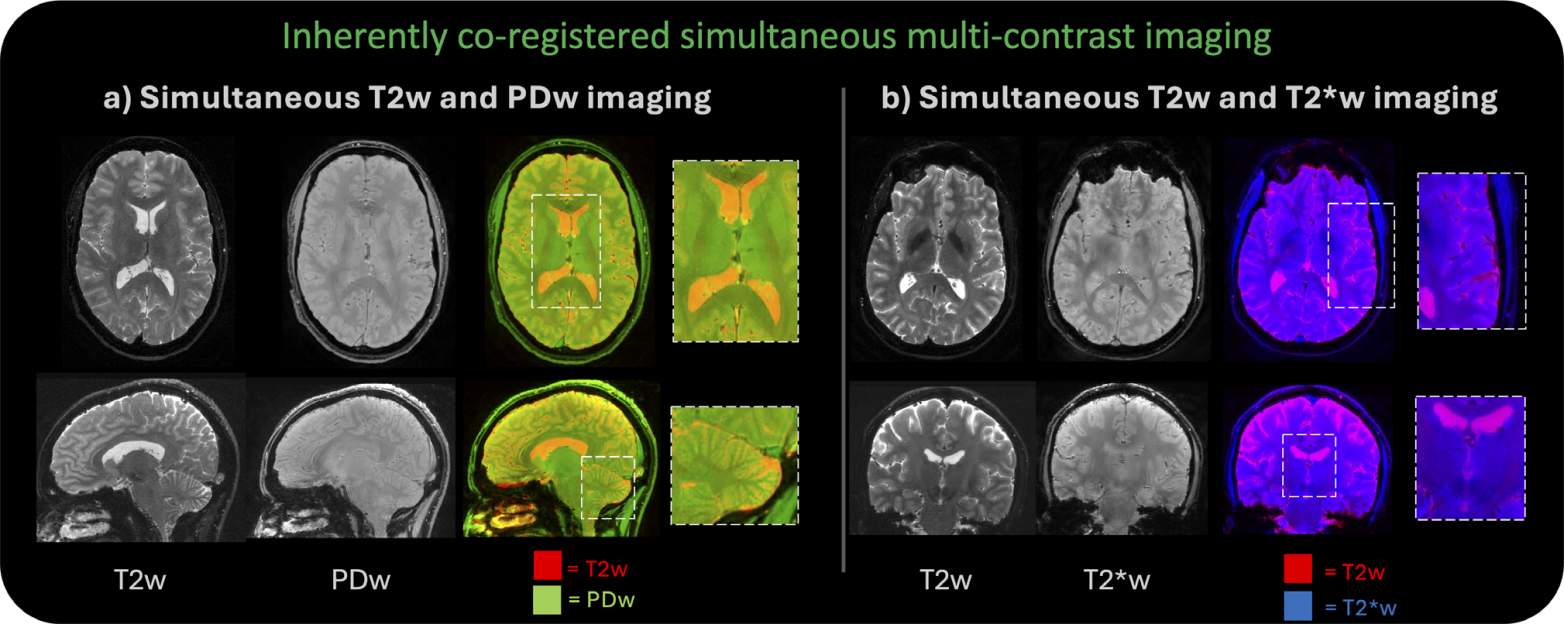
Inherently co‐registered simultaneous multi‐contrast imaging with the proposed LQ spiral sequence. (a) Simultaneous T2w and PDw imaging. Individual contrasts (left) and color overlays (red = T2w, green = PDw, right) demonstrate perfect spatial alignment of tissue boundaries. Insets highlight fine cortical and ventricular structures, showing consistent delineation across contrasts. (b) Simultaneous T2w and T2*‐weighted (T2w) imaging. Together, these results highlight the intrinsic co‐registration achieved within a single acquisition; notably, the absence of color fringes or “halo” artifacts at high‐contrast interfaces which would otherwise appear as doubled structural edges in the insets if misalignment were present confirms the robustness of this simultaneous sampling strategy.

Finally, Figure 9 compares the proposed LQ framework with conventional 3D FSE for high‐resolution volumetric imaging. At 800 μm isotropic resolution, spiral‐PRIME achieves lesion conspicuity, cortical delineation, and cerebellar detail comparable to or exceeding that of 3D FSE in less than half the scan time. Figure S5 shows results from an independent subject, confirming the reproducibility of these findings and further demonstrating that the proposed framework maintains consistent image quality advantages over 3D FSE across subjects.

**FIGURE 9:**
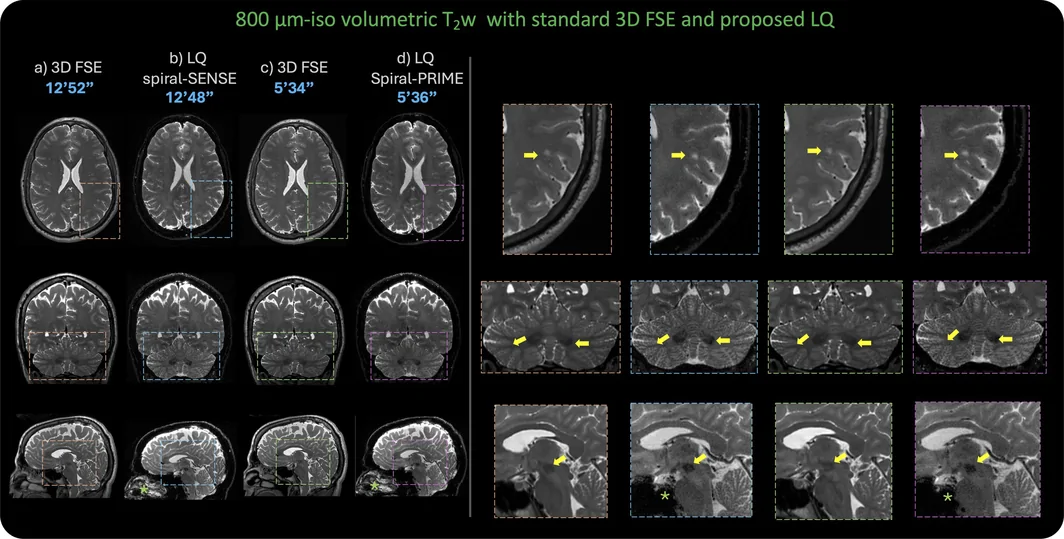
800 μm isotropic volumetric T2w imaging with conventional 3D FSE and the proposed LQ framework. Axial, coronal, and sagittal views are shown for (a) longer 3D FSE scan, (b) longer LQ with spiral‐SENSE, (c) shorter 3D FSE, and (d) shorter LQ with spiral‐PRIME. All scans were matched for resolution, FOV, TE, and TR, with scan times indicated. Insets highlight key regions with yellow arrows and asterisks. The 3D FSE images exhibit higher apparent SNR but with substantially reduced spatial resolution, which may arise from T2/T1‐related blurring associated with the long echo trains. In contrast, the spin‐echo–based LQ reconstructions preserve sharper tissue delineation and more consistent lesion conspicuity. Spiral readouts may exhibit mild T2*‐related blurring due to signal decay during the readout; however, the shorter spiral readout duration limits this effect. Spiral‐PRIME further improves cortical and cerebellar detail while suppressing undersampling noise, achieving image quality comparable to LQ with spiral‐SENSE in less than half the scan time. Green asterisks mark localized B0‐related drop‐out inherent to spiral readouts. Water‐only images are shown for LQ.

## 5 Discussion

This work introduces a LQ SE framework with spiral‐PRIME reconstruction that jointly tackles the two central limitations of spiral MRI, off‐resonance blurring and undersampling noise/artifacts. By integrating innovations in both acquisition and reconstruction, the proposed approach introduces several key advances: (i) efficient in‐plane sampling by distributing spiral arms to maximize k‐space coverage and disperse trajectory‐dependent inconsistencies as incoherent high‐frequency components (Figure S1), (ii) improved through‐plane robustness via sliding‐slice acquisition combined with a novel loop‐ordering strategy that eliminates repeated startup cycles and mitigates the appearance of motion artifacts (Figure 4), (iii) simultaneous multi‐contrast capability through additional gradient‐echo readouts that are inherently co‐registered without prolonging scan time (Figure 8). Notably, we advance the simultaneous multi‐contrast capability that was a hallmark of traditional SE imaging into the volumetric domain, and (iv) a unified ADMM‐based reconstruction (spiral‐PRIME) that performs joint spiral in‐out deblurring, fat–water separation, and 3D wavelet denoising, achieving substantial SNR gains while suppressing undersampling artifacts (Figures 5, 6, 7). Collectively, these elements establish LQ spiral imaging with spiral‐PRIME as a practical and efficient alternative to conventional 3D FSE for high‐resolution volumetric brain imaging (Figures 9 and S5).

Mirroring the LQ acquisition, spiral‐PRIME is inherently hybrid: slice‐wise CG projection enforces local data fidelity, while volumetric denoising and ADMM updates exploit 3D sparsity to suppress inter‐slice inconsistencies. This design effectively leverages the incoherent artifact structure generated by the acquisition, facilitating artifact mitigation through $\ell_{1}$ regularization. The impact is evident in Figures 5, 6, and 9: despite an undersampling factor of 2.33, spiral‐PRIME reconstructions are visually similar to fully sampled spiral‐SENSE reconstructions, with preserved lesion conspicuity, venous darkening, and fine detail (which theoretically requires a ∼50% SNR boost to match the reference). Reconstruction converged within six ADMM iterations, requiring approximately 2–3 min per iteration for dual‐trajectory data on a multi‐core CPU. While current processing takes several minutes per dataset, significant acceleration is achievable by porting the iterative CG solver and wavelet denoising steps to GPU architectures.

The SNR and CNR analyses in Figure 7 highlight tissue‐dependent benefits of spiral‐PRIME on dataset acquired using the protocol “US‐T2w/T2*w” shown in Table 1. For T2w, WM and GM show the largest SNR gains (≈30%–40%), while CSF and fat benefit less, consistent with baseline SNR: CSF is already bright and high‐SNR, leaving less room for improvement, whereas WM/GM gain more from wavelet denoising and joint fat–water modeling. T2*w exhibits substantially larger gains across all tissues, likely reflecting greater transform sparsity (strong vessel/edge content, reduced textural complexity), which wavelet regularization exploits more effectively. Nevertheless, even a ∼40% SNR improvement in WM/GM is clinically meaningful, enabling finer cortical delineation, improved gray–white matter contrast, and more reliable reformats at shorter scan times. CNR gains follow a similar trend, with the strongest improvements in GM–WM, followed by CSF–WM and CSF–GM. This suggests spiral‐PRIME not only reduces voxel‐wise variance but also enhances tissue boundaries, consistent with improved lesion conspicuity and venous detail. One important limitation of the pseudo‐replica SNR analysis is that it primarily reflects variance reduction and does not capture perceptual gains from suppressing incoherent undersampling residue; thus, our SNR gains likely underestimate the improvement in perceived image quality motivating clinical reader studies to quantify diagnostic impact.

Figure 9 compares the proposed LQ framework with conventional 3D FSE under both linear (spiral‐SENSE) and nonlinear (spiral‐PRIME) reconstructions, isolating acquisition physics from reconstruction effects. The linear‐to‐linear comparison confirms that sharpness gains originate from the single SE acquisition rather than the reconstruction. While 3D FSE exhibits higher apparent SNR, this largely reflects blur‐induced smoothing from the ∼861 ms echo trains required for volumetric efficiency. This near‐second‐long decay window causes irreversible point‐spread‐function broadening, smoothing cortical detail regardless of the acceleration factor or the number of averages (NEX). Furthermore, vendor‐enforced NEX = 2 constraints on our clinical scanner for axial orientation necessitated this comparison to ensure echo‐train stability. Importantly, a prior study [4] demonstrated that LQ achieves equivalent SNR efficiency to 3D FSE, supporting the interpretation that the higher apparent SNR of 3D FSE reflects blur‐related smoothing rather than true SNR efficiency gain. While reconstruction‐based approaches such as T2‐shuffling [25] are promising for compensating this inherent blurring in 3DFSE, the LQ framework addresses the problem at its physical source. By employing a pure single SE acquisition, LQ encodes structural information at a single, consistent T2 weighting and avoids the extensive signal decay intrinsic to long refocusing trains. Eliminating T2 decay during acquisition preserves sharp tissue boundaries and native contrast without relying on complex signal unmixing during reconstruction.

While spiral readouts are susceptible to B0 off‐resonance, this is a known effect explicitly modeled and corrected in our forward operator. Spiral readouts are susceptible to B0 off‐resonance effects; however, these effects are explicitly modeled and corrected in the forward operator. Spiral acquisitions may also exhibit T2* related blurring due to signal decay during the long readout. In the present implementation, the spiral readout duration is approximately 20 ms. Brain T2* values at 3T are heterogeneous and can span approximately 10‐70 ms, with substantially shorter values occuring in regions affected by strong susceptibility gradients. Therefore, while the readout duration is shorter than T2* in many brain tissues, it may be comparable to or longer than T2* in susceptibility‐affected regions, where T2*‐related blurring may be more pronounced. The extent of this effect is therefore expected to depend on tissue properties and local susceptibility conditions. Conversely, 3D FSE acquisitions may exhibit T2/T1‐related blurring arising from signal evolution during long echo trains. The extent and spatial distribution of this blurring depend not only on tissue relaxation properties, but also on implementation‐specific factors such as echo‐train duration, refocusing flip‐angle schedule, and ky–kz phase‐encoding ordering. In the implementation studied here, the echo train duration was approximately 861 ms. The relative impact of these relaxation‐related effects is therefore expected to be tissue dependent and sequence dependent. The qualitative observations shown in Figure 9 and reproduced across independent subjects (Figure S5) suggest that the proposed LQ framework preserves sharper structural delineation under the acquisition conditions evaluated in this study.

While the sliding‐slice LQ framework substantially improves through‐plane uniformity, subtle “Venetian‐blind” periodic intensity modulations remain visible in some regions, most notably the cerebellum, shown in Figure 9. These are attributed to non‐zero first‐order gradient moments (M1) in the refocusing and crushing pair during physiological motion, and M1‐nulled architectures are being developed to stabilize signal integrity [2]. Beyond these specific artifacts, some broader technical trade‐offs remain when comparing the proposed LQ‐spiral SE framework to clinical 3D‐FSE. Notably, spiral trajectories lack the “free” FOV provided by Cartesian frequency encoding, meaning any signal from anatomy outside the prescribed FOV will alias into the image and may necessitate a larger acquisition volume. Second, the current reconstruction framework relies on an accurate B0 field map to correct off‐resonance blurring. Unlike 3D FSE, which is largely insensitive to B0 inhomogeneity during readout, spiral imaging requires explicit modeling of this effect during reconstruction. In the initial implementation used in this study, acquisition of the B0 map required approximately 1.5 min. The calibration sequence has since been optimized and now requires only about 30 s. Nevertheless, this additional calibration step remains a practical requirement of the present implementation. Work is currently underway to develop deblurring approaches that estimate or compensate off‐resonance effects directly during reconstruction, which would eliminate the need for a separate field‐map acquisition. Finally, the inherent sensitivity of non‐Cartesian sampling to hardware imperfections requires rigorous calibration; however, in the clinical scanner used for this study, vendor‐provided gradient pre‐emphasis ensured that actual and nominal trajectories matched well in this study.

Looking ahead, clinical reader studies directly comparing spiral‐PRIME with both spiral‐SENSE and 3D FSE will be needed on a larger cohort including patients to clarify whether the sharper contrast preservation and reduced blurring in LQ translate into measurable improvements in lesion conspicuity, gray–white matter differentiation, and overall diagnostic confidence.

## 6 Conclusion

This work presents an LQ SE framework with spiral‐PRIME reconstruction that integrates novel sampling strategies and a hybrid model‐based reconstruction to simultaneously address motion, off‐resonance blurring, chemical‐shift, and undersampling artifacts and noise. The approach achieves multi‐contrast imaging, improved temporal efficiency, and substantial SNR gains compared to spiral‐SENSE, producing high‐resolution volumetric brain images comparable to or better than conventional 3D FSE in markedly shorter scan times. While these results highlight the technical feasibility and potential advantages of the proposed LQ framework with spiral‐PRIME, prospective clinical reader studies will be used to confirm diagnostic equivalence or superiority versus 3D FSE.

## Funding

This work was supported by the National Institute of Biomedical Imaging and Bioengineering, R01EB036009.

## Conflicts of Interest

James G. Pipe receives research support from GE Healthcare.

## Supporting information


**Table S1:** Quantitative evaluation of reconstruction quality across different sampling strategies. Peak signal‐to‐noise ratio (PSNR) and structural similarity index (SSIM) are reported for dual‐trajectory (spiral‐in–out) and single‐trajectory (spiral‐out) acquisitions at acceleration factors *R* = 2, 3, and 4. Dual‐trajectory sampling consistently outperforms single‐trajectory sampling, yielding higher PSNR and SSIM across all acceleration factors.
**Figure S1:** Effect of in‐plane sampling strategy on undersampling artifacts. (a) Fully sampled reference reconstruction. (b) *R* = 3 undersampling with the *same arm selection across all trajectories* produces structured aliasing artifacts in the water image (yellow arrows). (c) *R* = 3 undersampling with the *proposed in‐plane sampling* distributes arm selections differently across trajectories, dispersing inconsistencies as high‐frequency, pseudo‐random noise (yellow arrow). This strategy reduces coherent aliasing in the water channel and renders fat‐channel artifacts more noise‐like, demonstrating the improved incoherence of the proposed sampling design. Bottom row shows the corresponding spiral sampling patterns for each case.
**Figure S2:** Impact of ADMM penalty parameter $\rho_{0}$ and growth factor γ on reconstruction quality and convergence. (a–c) Quantitative evaluation of PSNR, SSIM, and residual versus iteration for different values of the initial penalty $\rho_{0}$, with growth factor fixed at γ=1.1. Small $\rho_{0}$ over‐enforces data consistency and degrades quality, whereas moderate values ($\rho_{0}\approx 1.0$) yield the best trade‐off between convergence and fidelity. (d–f) Convergence behavior for different growth factors γ, with $\rho_{0}=1.0$. A mild growth factor (γ≈1.1−1.3) accelerates convergence and improves final image quality compared with fixed‐ρ (γ=1.0), while excessive growth (γ≥1.4) destabilizes convergence. (g) Fully sampled CG reconstruction used as the reference. (h) Proposed reconstructions at selected iterations (Iter 1, 2, 3, 4, and 20). Insets (yellow boxes) highlight cortical detail on a 3× undersampled data. Artifacts are suppressed within the first 2–4 iterations, after which the reconstruction stabilizes and closely matches the fully sampled reference.
**Figure S3:** Representative T2*‐weighted images reconstructed using (a) spiral‐SENSE, (b) spiral‐PRIME with slice‐wise (2D) wavelet denoising, and (c) spiral‐PRIME with 3D wavelet denoising. The spiral‐SENSE reconstruction retains fine detail but is affected by high noise. Slice‐wise wavelet denoising reduces noise but introduces slice‐dependent artifacts and irregularities (red arrows). In contrast, 3D wavelet denoising achieves consistent noise suppression across slices and preserves structural fidelity without introducing spurious slice‐level artifacts.
**Figure S4:** Voxel‐wise statistics from pseudo‐replica ensembles reconstructed with spiral SENSE‐Recon and spiral‐PRIME. (a) Mean of 32 pseudo‐replica reconstructions demonstrates consistent anatomy across both methods, confirming reconstruction stability. (b) Voxel‐wise standard‐deviation maps highlight substantially reduced noise variance with spiral‐PRIME relative to spiral SENSE‐Recon, consistent with the SNR gain reported in the main text. These results illustrate that the improvement arises from reduced noise amplification rather than altered mean signal.
**Figure S5:** 800 μm isotropic volumetric T₂‐weighted imaging with conventional 3D FSE and the proposed LQ spiral‐PRIME, matched for scan time (∼5′36″). Axial, and coronal reformats are shown for (a) 3D FSE and (b) LQ spiral‐PRIME. Insets (right) highlight representative regions with yellow arrows, illustrating sharper cortical delineation, improved deep gray matter contrast, and clearer cerebellar folia detail with spiral‐PRIME compared to 3D FSE, which likely suffers from T2 blurring and reduced contrast due to long echo trains.

## Data Availability

The Spiral‐PRIME reconstruction code and a representative sample dataset are available at https://github.com/gpilab/spiral‐prime; additional data are available on reasonable request.

## Appendix A ADMM Reconstruction Formulation

The joint water‐fat reconstruction derived in Equation (8) was solved using ADMM. Let $x=\left[x_{w},x_{f}\right]$, $z=\left[z_{w},z_{f}\right]$, $u=\left[u_{w},u_{f}\right]$. With forward operator $\tilde{E}$ and data d,

(A1)
$$\underset{x,z}{\min\frac{1}{2}}\left\|\tilde{E}x-d\right\|\begin{array}{c} 2\\ 2 \end{array}+\sum_{c\in \left\{w,f\right\}}\lambda_{c}{\left\|\Psi z_{c}\right\|}_{1}\;s.t.\;x=z$$

where Ψ is a 3D wavelet transform.

The augmented Lagrangian is

(A2)
$$L_{p}\left(x,z,u\right)=\frac{1}{2}\left\|\tilde{E}x-d\right\|\begin{array}{c} 2\\ 2 \end{array}+\sum_{c}\lambda_{c}{\left\|\Psi z_{c}\right\|}_{1}+\frac{\rho }{2}\left\|x-z+u\right\|\begin{array}{c} 2\\ 2 \end{array}$$

Each iteration k consists of:

1. Projection step (x‐update):
  (A3)
  $$\left({\tilde{E}}^{H}\tilde{E}+\rho I\right)x^{k+1}=\tilde{E}d+\rho \left(z^{k}-u^{k}\right)$$
  Solved by conjugate gradient independently for each slice.
2. Denoising step (z‐update):
  (A4)
  $$z_{c}^{k+1}=\Psi^{-1}(\text{soft}\left(\Psi \left(x^{k+1}\right))+u^{k},\lambda_{c}^{k}\right),c\in \left\{w,f\right\}$$
  Implemented as a 3D wavelet soft thresholding with cycle spinning. Soft thresholds are sub‐band specific.
3. Dual update:

(A5)
$$u^{k+1}=u^{k}+x^{k+1}-z^{k+1}$$

4 Penalty scheduling:
  The penalty parameter ρ was initialized at $\rho_{0}$ and increased geometrically across iterations,
  (A6)
  $$\rho^{k+1}=\gamma \rho^{k},\gamma >1$$
  To progressively enforce data consistency. The effective soft threshold in the denoising was scaled as
  (A7)
  $$\lambda_{c}^{k}=\frac{\rho_{0}}{\rho^{k}}{\beta \theta }_{c},0<\beta <1$$
  where β is a user‐defined damping factor to set the desired denoising level and $\theta_{c}$ is a wavelet band‐wise soft threshold obtained using SureShrink on $x^{0}$.
