# Supplementary material for: Localized Quadratic RF Encoded Spin‐Echo With Spiral‐PRIME Reconstruction: A Practical Alternative to 3D FSE for High‐Resolution Volumetric Brain MRI
Source: Magn Reson Med. 2026 Aug 18;96(6):2615–28. doi: 10.1002/mrm.70476 (PMC13619862; doi:10.1002/mrm.70476)
Supplement: Supplementary file 1 [file MRM-96-2615-s001.docx]

| **Contrast** | **Acceleration Factor** | **PSNR (dB)** | **SSIM** |
| --- | --- | --- | --- |
| **Dual trajectory**  **(spiral- n-out)** | 2 | 35.02 | 0.88 |
|  | 3 | 33.20 | 0.85 |
|  | 4 | 31.05 | 0.82 |
| **Single trajectory**  **(Spiral-out)** | 2 | 32.27 | 0.87 |
|  | 3 | 30.72 | 0.84 |
|  | 4 | 28.84 | 0.80 |

**Supplementary Table 1**. Quantitative evaluation of reconstruction quality across different sampling strategies. Peak signal-to-noise ratio (PSNR) and structural similarity index (SSIM) are reported for dual-trajectory (spiral-in–out) and single-trajectory (spiral-out) acquisitions at acceleration factors R = 2, 3, and 4. Dual-trajectory sampling consistently outperforms single-trajectory sampling, yielding higher PSNR and SSIM across all acceleration factors.

**Supporting Figure S1.** Effect of in-plane sampling strategy on undersampling artifacts. (a) Fully sampled reference reconstruction. (b) R=3 undersampling with the *same arm selection across all trajectories* produces structured aliasing artifacts in the water image (yellow arrows). (c) R=3 undersampling with the *proposed in-plane sampling* distributes arm selections differently across trajectories, dispersing inconsistencies as high-frequency, pseudo-random noise (yellow arrow). This strategy reduces coherent aliasing in the water channel and renders fat-channel artifacts more noise-like, demonstrating the improved incoherence of the proposed sampling design. Bottom row shows the corresponding spiral sampling patterns for each case.

 **Supporting Figure S2.** Impact of ADMM penalty parameter $\rho_{0}$ and growth factor $\gamma$ on reconstruction quality and convergence. (a–c) Quantitative evaluation of PSNR, SSIM, and residual versus iteration for different values of the initial penalty $\rho_{0}$, with growth factor fixed at $\gamma=1.1$. Small $\rho_{0}$ over-enforces data consistency and degrades quality, whereas moderate values ($\rho_{0}\approx1.0$) yield the best trade-off between convergence and fidelity. (d–f) Convergence behavior for different growth factors $\gamma$, with $\rho_{0}=1.0$. A mild growth factor ($\gamma\approx1.1\text{–}1.3$) accelerates convergence and improves final image quality compared with fixed-$\rho$ ($\gamma=1.0$), while excessive growth ($\gamma\geq1.4$) destabilizes convergence. (g) Fully sampled CG reconstruction used as the reference. (h) Proposed reconstructions at selected iterations (Iter 1, 2, 3, 4, and 20). Insets (yellow boxes) highlight cortical detail on a 3X undersampled data. Artifacts are suppressed within the first 2–4 iterations, after which the reconstruction stabilizes and closely matches the fully sampled reference.

**Supporting Figure S3.** Representative T2*-weighted images reconstructed using (a) spiral-SENSE, (b) spiral-PRIME with slice-wise (2D) wavelet denoising, and (c) spiral-PRIME with 3D wavelet denoising. The spiral-SENSE reconstruction retains fine detail but is affected by high noise. Slice-wise wavelet denoising reduces noise but introduces slice-dependent artifacts and irregularities (red arrows). In contrast, 3D wavelet denoising achieves consistent noise suppression across slices and preserves structural fidelity without introducing spurious slice-level artifacts.

**Supplementary Figure S4.** Voxel-wise statistics from pseudo-replica ensembles reconstructed with spiral-SENSE Recon and spiral-PRIME. (a) Mean of 32 pseudo-replica reconstructions demonstrates consistent anatomy across both methods, confirming reconstruction stability. (b) Voxel-wise standard-deviation maps highlight substantially reduced noise variance with spiral-PRIME relative to spiral SENSE-Recon, consistent with the SNR gain reported in the main text. These results illustrate that the improvement arises from reduced noise amplification rather than altered mean signal.

**Supplementary Figure S5.** 800 µm isotropic volumetric T₂-weighted imaging with conventional 3D FSE and the proposed LQ spiral-PRIME, matched for scan time (~5’36”). Axial, and coronal reformats are shown for (a) 3D FSE and (b) LQ spiral-PRIME. Insets (right) highlight representative regions with yellow arrows, illustrating sharper cortical delineation, improved deep gray matter contrast, and clearer cerebellar folia detail with spiral-PRIME compared to 3D FSE, which likely suffers from T2 blurring and reduced contrast due to long echo trains.
